# Tumor-Educated Platelets and Angiogenesis in Glioblastoma: Another Brick in the Wall for Novel Prognostic and Targetable Biomarkers, Changing the Vision from a Localized Tumor to a Systemic Pathology

**DOI:** 10.3390/cells9020294

**Published:** 2020-01-25

**Authors:** Rolando Campanella, Laura Guarnaccia, Chiara Cordiglieri, Elena Trombetta, Manuela Caroli, Giorgio Carrabba, Nicla La Verde, Paolo Rampini, Chiara Gaudino, Antonella Costa, Sabino Luzzi, Giovanna Mantovani, Marco Locatelli, Laura Riboni, Stefania Elena Navone, Giovanni Marfia

**Affiliations:** 1 Laboratory of Experimental Neurosurgery and Cell Therapy, Neurosurgery Unit, Fondazione IRCCS Ca’ Granda Ospedale Maggiore Policlinico, 20122 Milan, Italy; 2 Department of Clinical Sciences and Community Health, University of Milan, 20122 Milan, Italy; 3 Imaging Facility, National Institute for Molecular Genetics (INGM), 20122 Milan, Italy; 4 Flow Cytometry Service, Clinical Laboratory, Foundation IRCCS Ca’ Granda Ospedale Maggiore Policlinico, 20122 Milan, Italy; 5 Oncology Unit, ASST Sacco Hospital, 20157 Milan, Italy; 6 Department of Neuroradiology, Fondazione IRCCS Ca’ Granda Ospedale Maggiore Policlinico, University of Milan, 20122 Milan, Italy; 7 Neurosurgery Unit, Department of Clinical-Surgical, Diagnostic and Pediatric Sciences, University of Pavia, 27100 Pavia, Italy; 8 Neurosurgery Unit, Department of Surgical Sciences, Fondazione IRCCS Policlinico San Matteo, 27100 Pavia, Italy; 9 Endocrinology Unit, Fondazione IRCCS Ca’ Granda, Ospedale Maggiore Policlinico, 20122 Milan, Italy; 10 Department of Medical-Surgical Physiopathology and Transplantation, University of Milan, 20122 Milan, Italy; 11 Department of Medical Biotechnology and Translational Medicine, LITA-Segrate, University of Milan, 20090 Milan, Italy

**Keywords:** glioblastoma, platelets, sphingosine-1-phosphate, angiogenesis

## Abstract

Circulating platelets (PLTs) are able to affect glioblastoma (GBM) microenvironment by supplying oncopromoter and pro-angiogenic factors. Among these mediators, sphingosine-1-phophate (S1P) has emerged as a potent bioactive lipid enhancing cell proliferation and survival. Here, we investigated the effect of “tumor education”, characterizing PLTs from GBM patients in terms of activation state, protein content, and pro-angiogenic potential. PLTs from healthy donors (HD-PLTs) and GBM patients (GBM-PLTs) were collected, activated, and analyzed by flow cytometry, immunofluorescence, and Western blotting. To assess the pro-angiogenic contribution of GBM-PLTs, a functional cord formation assay was performed on GBM endothelial cells (GECs) with PLT-releasate. GBM-PLTs expressed higher positivity for P-selectin compared to HD-PLTs, both in basal conditions and after stimulation with adenosine triphosphate (ADP) and thrombin receptor activating peptide (TRAP). PLTs showed higher expression of VEGFR-1, VEGFR-2, VWF, S1P, S1PR1, SphK1, and SPNS. Interestingly, increased concentrations of VEGF and its receptors VEGFR1 and VEGFR2, VWF, and S1P were found in GBM-PLT-releasate with respect to HD-PLTs. Finally, GBM-PLT-releasate showed a pro-angiogenic effect on GECs, increasing the vascular network’s complexity. Overall, our results demonstrated the contribution of PLTs to GBM angiogenesis and aggressiveness, advancing the potential of an anti-PLT therapy and the usefulness of PLT cargo as predictive and monitoring biomarkers.

## 1. Introduction

Glioblastoma (GBM) is the most frequent and deadliest primary human brain tumor, as patients rarely survive beyond 14 months, with a 5-year survival rate of around 5% [1]. GBM has virtually no effective treatment, representing a considerable therapeutic challenge for which novel paradigms should be considered. GBM is characterized by rapid progression, invasiveness, intense angiogenesis, resistance to radio- and chemotherapies, and a high frequency of relapse. As defined in the Stupp protocol, the current GBM standard of care includes surgical resection followed by radiotherapy with concomitant and adjuvant temozolomide (TMZ) [2]. Despite these aggressive approaches, most patients suffer recurrence after less than one year. The therapy resistance of GBM is mostly due to a subpopulation of tumorigenic stem-like cells, known as GBM stem cells (GSCs), which are self-renewing, pluripotent, highly proliferative, and genetically unstable. GSCs hierarchically drive tumor progression and recurrence [3]. Upon pro-tumorigenic stimuli, GSCs can transdifferentiate into GBM endothelial cells (GECs), to directly contribute to the perivascular niche [4]. GECs trigger pro-angiogenic factor production and the formation of abnormal blood vessels to sustain GBM growth [5]. Importantly, transdifferentiated GECs can also acquire resistance and genetic mutations [6]. The communication between GECs and GSCs is a two-way exchange in which, aside from direct cell–cell communication, they can also bidirectionally exchange signals, including nucleic acids, proteins, and metabolites, thereby creating a permissive tumor microenvironment (TME). Among TME signals, bioactive membrane lipids, such as sphingolipids, may play an important role in promoting tumorigenesis and cancer malignancy. Indeed, sphingosine-1-phosphate (S1P) is able to stimulate cellular processes strictly related to cancer, such as proliferation, invasion, survival, and angiogenesis [7]. The biosynthesis of S1P occurs during complex sphingolipid catabolism via the conversion of ceramide (Cer) to sphingosine (Sph), which is then phosphorylated to S1P by Sph kinases (SphK1/2) [8]. The secretion of S1P out of the cell is mediated by the transporter spinster homology protein 2 (Spns2), which belongs to the major facilitator superfamily (MFS). As an extracellular signal, S1P exerts its effects by binding to five specific G protein-coupled receptors (S1PR1–5) located in the plasma membrane [8]. S1P also plays pleiotropic functions in gliomagenesis. In particular, rapidly proliferating GSCs exhibit fast conversion of Sph to S1P, perform rapid degradation of newly synthesized Cer, and release a 10-fold higher amount of S1P in the extracellular milieu compared to slow-proliferating GSCs [9]. Interestingly, previous studies reported that GECs are also well equipped to produce and release S1P and, when co-cultured with GBM cells, GECs exhibited increased SphK2 expression and activity, with significant S1P secretion enhancement. In turn, in an autocrine/paracrine manner, extracellular S1P stimulates GBM cell growth and GEC migration and tubule formation in an S1PR1/S1PR3-dependent trend [10]. In GBM, GECs undergo activation, thereby causing an increased exposition of von Willebrand Factor (VWF) on their surface. VWF, a multimeric adhesive glycoprotein, is mainly secreted from Weibel Palade Bodies (WPBs) of GECs. Increased plasma levels of VWF antigen (VWF:Ag) have been found in GBM patients, with higher VWF plasma levels correlating with shorter survival, rendering VWF:Ag levels a putative circulating prognostic factor [11]. The mechanisms involved in VWF exocytosis are poorly understood, but recent observations revealed that S1P is able to trigger increased WPB secretion and endothelial barrier disruption [12]. Moreover, VWF may protect cancer cells from immune cells and mediate platelet (PLT) adhesion to vascular damage sites [13]. At the site of injury, PLTs degranulate, releasing pro-angiogenic factors such as VWF, vascular endothelial growth factor (VEGF), and S1P. Interestingly, PLTs were originally suggested to be the prime source of plasmatic S1P because of their high SphK activity [14] and lack of S1P lyase [15]. Thus, it is reasonable to consider PLTs as functional circulating carriers of S1P [16,17,18] acting in a dual process: (i) accumulating huge reservoirs of biomolecules, sequestering tumor-derived factors/signals (e.g., growth factors, cytokines, nucleic acids), and thus developing into “tumor-educated” PLTs (TEPs) [19], and (ii) releasing microparticles shuttled to tumors to regulate both anti- and pro-tumorigenic gene expression programs. Previous results reported that PLTs from GBM patients showed significantly elevated concentrations of VEGF when compared to healthy subjects, with a negative correlation between PLT-released VEGF and patient prognosis [20]. In this study, we present our preliminary data on PLT characterization from GBM patients in terms of activation state, protein content, and pro-angiogenic effects, in order to investigate their role as pro-tumoral carriers.

## 2. Materials and Methods

### 2.1. Patient Recruitment

GBM patients (*n* = 10) were recruited during hospitalization at the Neurosurgery Unit of Fondazione IRCCS Ca’ Granda Ospedale Maggiore Policlinico. The Institutional Review Board approved the protocol and all patients provided informed consent. All research was performed in accordance with relevant guidelines and regulations. The inclusion criteria for patient enrolment were as follows: (i) aged between 18–75 years; (ii) Karnofsky performance status (KPS) >70; (iii) confirmed diagnosis of GBM; (iv) informed consent signed. The exclusion criteria were as follows: (i) presence of another primary tumor or proven metastasis; (ii) concomitant life-threatening disease; (iii) pathology affecting the blood circulation and the coagulation system. Demographic, clinical, and molecular characteristics of the patients are listed in Table 1.

### 2.2. Tumor Sample Collection and Processing

Tissue samples from patients who underwent surgical resection of GBM at the Neurosurgery Unit of Fondazione IRCCS Ca’ Granda Ospedale Maggiore Policlinico were collected. Tumor specimens were washed in D-PBS (Euroclone, Milan, Italy) and suspended in DMEM/F12 (ThermoFisher, Waltham, Massachusetts, USA) containing 1% penicillin/streptomycin. At the Laboratory of Experimental Neurosurgery and Cell Therapy, tissues were processed by fine mincing with a surgical scalpel in D-PBS and antibiotic solution. After shredding, samples were enzymatically digested with 0.625 Wu/mL Liberase Blendzyme 2 (Roche, Mannheim, Germany) for 1 h at 37 °C [5,21].

### 2.3. GEC Isolation and Characterization

The tissue suspension was filtered with a 0.22 µm pore-size filter to obtain a monocellular cell suspension. Cells were plated into a 25 cm^2^ flask coated with bovine type I collagen (BD Biosciences, Milan, Italy) and cultured in endothelial proliferation medium, EndoPM, at 37 °C in 5% CO_2_ and 5% O_2_, according to published protocols [5,21,22], in order to isolate the endothelial cell population (ECs). The media was changed 1–2 times a week and passaged at a split ratio of 1:4 every 14 days [5,9].

### 2.4. Blood Sample Collection

Blood samples from GBM patients (*n* = 6) were collected from patients undergoing surgery for GBM resection. The blood was withdrawn in the presence of 3.2% Na-citrate the day before surgery after signed consent. Blood from healthy donors (HD, *n* = 4) was also obtained after informed consent. The enrolled subjects did not take aspirin or other nonsteroidal anti-inflammatory drugs for at least 10 days before the blood draw [20].

### 2.5. PLT Preparation and Stimulation

The whole blood was centrifuged at 150× *g* for 15 min to obtain platelet-rich plasma (PRP). The PRP was transferred into a new tube with ACD anticoagulant buffer 1:10 (6.25 g sodium citrate, 3.1 g citric acid anhydrous, 3.4 g glucose, pH 6.5 in 250 mL H_2_O), centrifuged (600× *g*, 5 min) and the resulting platelet-poor plasma (PPP) was stored at −20 °C. PLTs were then washed twice with HEPES-Tyrode’s buffer (134 mM sodium chloride, 12 mM sodium bicarbonate, 2.9 mM potassium chloride, 0.34 mM sodium phosphate monobasic, 1 mM magnesium chloride, 5 mM HEPES, 5 mM glucose, 1% BSA, pH 7.4), counted, and resuspended to produce a final concentration of 3 × 10^8^ platelets/mL. The PLTs were then incubated with adenosine diphosphate (ADP) at a concentration of 10 µM or thrombin receptor activating peptide (TRAP) at a concentration of 5 μM for 20 min at 37 °C to induce stimulation. Finally, the PLTs were centrifuged at 300× *g* for 5 min, and the supernatant containing the stimulated PLT-releasate was stored at −20 °C [20].

### 2.6. Flow Cytometry Analysis

Stimulated and non-stimulated PLTs (S-PLTs and NS-PLTs, respectively) were analyzed by flow cytometry for the expression of CD62P, a marker of activated PLTs. After incubation with FITC-conjugated CD62P, analysis was conducted using FACS Canto II flow cytometer and FACSDivasoftware (BD Bioscience, version 5.0) [20]. Forward- versus side-scatter (FSC-A vs. SSC-A) gating was used to identify intact PLTs based on size and granularity.

### 2.7. Immunofluorescence Analysis

NS-HD-PLTs, NS-GBM-PLTs, and S-GBM-PLTs (stimulated with thrombin at 0.1 U/mL, as previously described [20]) from 3 GBM patients and 2 age- and gender-matched HDs were seeded (1× 10^6^ PLTs/well) onto a µ-plate 96-well Black for optimized fluorescence-based imaging of the fixed cells. The PLTs were fixed in 8% paraformaldehyde 8for 20 min at room temperature (RT), washed twice with D-PBS, and incubated with 0.1 M glycine to quench autofluorescence. Then, the PLTs were treated with PBS + 0.25% Triton X-100 to permeabilize the cell membranes and then blocked in PBS + 5% BSA for 30 min at RT. Incubation with primary antibodies (AB-I) diluted in blocking buffer was performed overnight at 4 °C. The following AB-I were used: anti-VEGFR1 (ThermoFisher), anti- VEGFR2 (Abcam, Cambridge, UK), anti-VWF (ThermoFisher), anti-S1P (Sonepcizumab, Creative Biolabs, Shirley, New York, USA), anti-S1PR1 (Abcam), anti-SphK1 (Abcam), anti-SPNS (Abcam), anti-phalloidin, and anti-P-selectin (SantaCruz Technology). The following day, the AB-I were removed and, after three washes with PBS + 0.1% BSA, AB-II were added for 1h at RT [5,21,22]. Immunolabeling was acquired using a high-resolution Nikon Ti spinning disk microscope (Nikon Instruments, Florence, Italy) equipped with a CREST-optics spinning disk head, a VCS structure illumination module for super-resolution (CREST-Optics, Rome, Italy), and Andor cameras for resolution (Andor Zyla, Andor Technology, Oxford Instruments, Oxford, UK) and quantum efficiency (Andor Technology, Oxford Instruments, Oxford, UK). A TIRF 100× objective (NA 1.49) (Nikon Instruments, Florence, Italy) was used to acquire both spinning disk confocal images (over a 3-micron Z-stack with a 0.2-micron Z-step) and for structure illumination super-resolution acquisition. The Richardson–Lucy 3D deconvolution algorithm (NIS-Elements V.5.2.11) was used to deconvolve the spinning disk confocal images, whereas specific VCS-Studio algorithms (CREST Optics, Rome, Italy) were employed for correct structure illumination reconstruction. High resolution imaging was performed on all stained wells using the whole-well approach though large-mosaic rendering during high-magnification acquisition in order to obtain a complete field of view (FOV) of the whole well for each instance of double-IF labeling. Acquisitions of all wells and different labeling instances were performed using the same experimental parameters, the same set-up with similar LED excitation power (10–20% for all LED lines at 470, 555, and 640 nm), and the same detection parameters in terms of the spinning disk wavelength band-filters and EM-CCD camera read-out rates (50 msec exposure plus 100 EM-gain at 30 MHz frequency); processing, segmentation, and image quantifications were conducted simultaneously on all acquired FOVs. The processed images were quantified using ad-hoc prepared pipelines of image segmentation and quantification, employing both GA2 and GA3 modules of NS-Elements digital imaging analysis software (Nikon Instruments, Florence, Italy). In detail, the platelets were morphologically segmented and labeling positivity was used to both quantify the signal intensity and classify the objects for single and double labeling. Quantified image analysis parameters from all FOVS were subsequently plotted on the same graphs, thus allowing observation of the homogeneity and data distribution from the complete set of samples used for the IF assay.

### 2.8. Western Blot

A Western blot was performed to assess the concentration of pro-angiogenic proteins in the PLT-releasate of 4 HDs and 6 GBMs. Specifically, the protein amount was measured by Pierce Detergent Compatible Bradford Assay Kit (Thermo Fisher Scientific). An equal amount of protein (50 µg) was separated in Bolt 10% Bis-Tris Plus Gel (Thermo Fisher Scientific) in a Mini Gel Tank (Thermo Fisher Scientific) and transferred onto nitrocellulose iBlot 2 Transfer Stacks using the iBlot 2 Dry Blotting System (Thermo Fisher Scientific). After transfer, the membrane was blocked in Tris-buffered saline/Tween 20 + 5% milk solution and incubated separately with anti-GAPDH (SantaCruz Biotechnology), anti-VEGF (Abcam), anti-VEGFR2 (Abcam), anti-VWF (Thermo Fisher Scientific), or anti-S1P (Sonepcizumab, Creative Biolabs) overnight at 4 °C. After incubation with HRP-labeled secondary antibody (Invitrogen, Carlsbad, California, USA), the protein bands were scanned using SuperSignal West Femto PLUS Chemiluminescent Substrate (Thermo Fisher Scientific) and detected by iBright5000 ChemiDoc XRSþ (Thermo Fisher Scientific). Densitometric analyses were performed using ImageJ [21].

### 2.9. Tube-Formation Assay

To assess the effect of PLT-releasate on angiogenesis, GECs (1 × 10^4^/well) were seeded onto a μ-plate angiogenesis 96-well (Ibidi), which was pre-coated with 12.5 mg/mL Matrigel (BD Bioscience) and cultured at 37 °C in 5% CO_2_ and 5% O_2_ in the presence or absence of PLT-releasate diluted at 1:10 in EndoPM. The GECs cultured in EndoPM + HEPES-Tyrode buffer (1:10) were used as the untreated control, referred to as the basal medium (BM). Cord formation was monitored with an inverted Eclipse Ti-E microscope (Nikon Instruments, Florence, Italy), which was equipped with a high resolution cSMOS camera (Andor Zyla, Andor Technology, Belfast, UK) and NIS_Elements 4.51 software, using differential interference contrast (DIC). After 24 h of incubation, five random images were acquired and analyzed using the “Angiogenesis Analyzer” plugin in ImageJ [5,21,22].

### 2.10. Statistical Analysis

All analyses were performed with GraphPad (Prism 8). Cytofluorimetric parameters were analyzed using the Mann–Whitney *U* test for independent samples (HD vs. GBM) and by the Kruskall–Wallis test for the analysis of cord formation (BM and PLT-releasate from HD- and GBM-PLTs). When significant differences were detected, Dunnet post-hoc comparisons against the control group were made. Differences were considered statistically significant at *p* < 0.05. Values were expressed as the mean ± SD of at least 3 independent experiments.

## 3. Results

### 3.1. GBM-PLTs Show an Activated State

**Figure 1:**
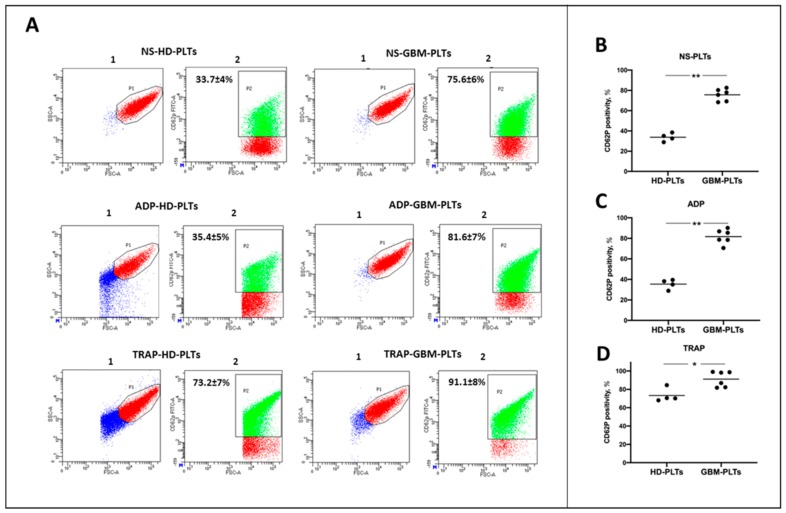
Glioblastoma platelets (GBM-PLTs) show a higher activated state compared to healthy donor (HD)-PLTs. (**A**) Representative dot plots from flow cytometry acquisition performed on HD- and GBM-PLTs under basal conditions and after stimulation with 10 µM adenosine triphosphate (ADP) and 5 µM thrombin receptor activated peptide (TRAP). NS: not stimulated. Plot 1 represents the first analysis performed according to side-scatter (SSC-A) and forward-scatter (FSC-A); blue cells represent PLTs with compromised scatter, i.e., cell debris, whereas red cells represent intact PLTs, which were further analyzed. In Plot 2, the red cells represent CD62P-negative PLTs, while the green cells are CD62P-positive. CD62P-expression levels were expressed as percentages and indicated in each box. (**B–D**) Platelet counts and platelet activation were determined by flow citometry analysis for CD62P in HD- and GBM-PLTs in the resting state (**B**) (NS) after ADP (**C**) and TRAP (**D**) stimulation. The expression of CD62P was significantly higher in GBM-PLTs both under basal conditions and after stimulation with ADP and TRAP. * *p* < 0.05, ** *p* < 0.01 versus healthy donors (HD).

Flowcytometric analyses were performed to determine the state of activation of HD- and GBM-PLTs. PLTs isolated from GBM patients expressed higher levels of CD62P (75.6% ± 6%) compared to HD-PLTs (33.7% ± 4%). When GBM-PLTs were treated with 10 µM ADP, we noticed that CD62P expression did not change in the HD-PLTs (35.4% ± 5%), and only a slight increase was detected in the GBM-PLTs (81.6% ± 7%) (Figure 1A). Contrarily, after the stimulation of PLTs with TRAP, which is considered to be the strongest platelet activator via protease-activated receptors, HD-PLTs reached a CD62P-positivity percentage of 73.2% ± 7%, whereas GBM-PLT stimulation resulted in an almost total activation of 91.1% ± 8%. Representative flow cytometric plots for the detection of platelet activation and CD62P positivity quantification are reported in in Figure 1B–D. Notably, the blue cells represent PLTs with compromised scatter, indicating damaged PLTs and cell debris; these were excluded from the analysis for CD62P-positivity.

### 3.2. GBM-PLTs Express and Release More Pro-Angiogenic and Pro-Tumoral Markers than HD-PLTs

**Figure 2:**
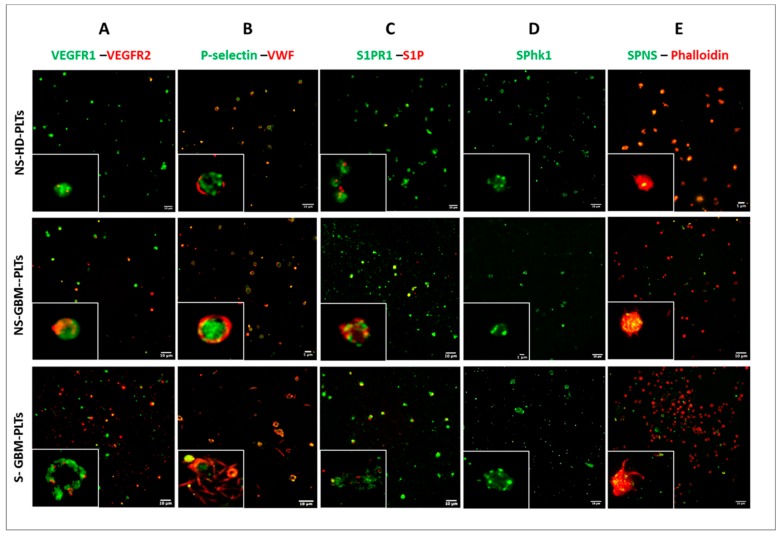
Localization of pro-angiogenic and pro-tumoral molecules in GBM-PLTs and HD-PLTs by immunophenotypic analysis. Representative images of immunofluorescence analyses conducted on non-stimulated (NS)-HD-PLTs, NS-GBM-PLTs, and stimulated (S)-GBM-PLTs. Immunolabeling was performed for (**A**) VEGFR1 and VEGFR2, (**B**) P-selectin and VWF, (**C**) S1PR1 and S1P, (**D**) SphK1, (**E**) SPNS and Phalloidin. The white box contains an enlargement of the image. Scale bar: 10 μm.

**Figure 3:**
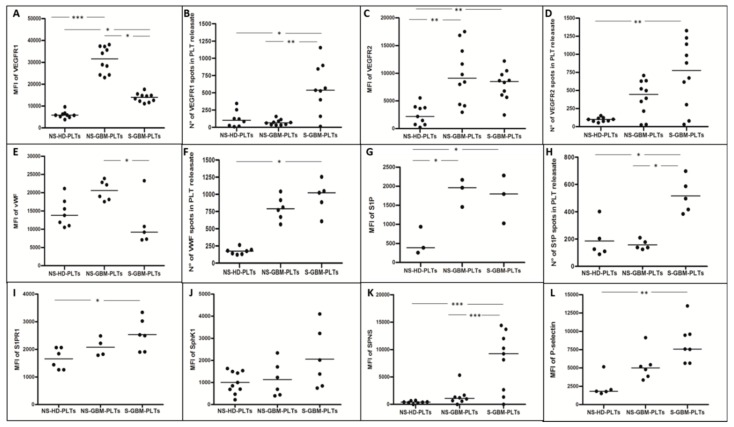
GBM-PLTs expressed and released pro-angiogenic and pro-tumoral molecules compared to HD-PLTs. Data from (**A**)VEGFR1, (**C**)VEGFR2, (**E**) VWF, (**G**) S1P, (**I**) S1PR1, (**J**) SPhK1, (**K**) SPNS, and (**L**) P-selectin labelings of PLTs from HD and GBM patients in non-stimulated (NS-) and stimulated (S-) conditions. Image analysis through ad-hoc-created pipeline of binary segmentation and object quantification to detect and analyze the specific mean fluorescence intensity (MFI) of PLTs in an 18-mm^2^ field of view (FOV). The number of spots of (**B**) VEGFR1, (**D**) VEGFR2, (**F**)VWF, and (**H**) S1P were detected and counted with the same pipeline of binary segmentation in the PLT-releasate. * *p* < 0.05, ** *p* < 0.01, *** *p* < 0.001 versus healthy donors (HD).

Immunofluorescence revealed intense positivity of GBM-PLTs to pro-tumoral and pro-angiogenic factors compared to HD-PLTs. GBM-PLTs showed higher expression of VEGF- signaling, VWF, and P-selectin, which are markers of angiogenesis and endothelial activation. Higher expression levels of both VEGFR1 and VEGFR2 were detected in GBM-PLTs compared to HD-PLTs; interestingly, after stimulation, the localization of VEGF receptors moved from the cytoplasm to the cell membrane (Figure 2, column A). Indeed, the quantification in the field of view (FOV) of the mean fluorescence intensity (MFI) for VEGFR1 showed a significantly more intense signal in GBM-PLTs compared to HD-PLTs, with decreased signal intensity in GBM-PLTs after stimulation (Figure 3A). In turn, VEGFR1 MFI was significantly higher in the releasate in S-GBM-PLTs with respect to NS-GBM- and HD-PLTs (Figure 3B). The same trend was also observed for VEGFR2, where PLTs showed a significantly more intense signal in GBM-PLTs in both the resting and stimulated states compared to HDs (Figure 3C). Like VEGFR1, the VEGFR2 MFI was also significantly higher after PLT stimulation in GBM patients compared to HD subjects (Figure 3D). This condition may be explained by the fact that after activation, PLTs were more prone to receive extracellular signals and to initiate intracellular signaling pathways. The immunolabeling for VWF revealed a higher positivity in GBM-PLTs than in HD-PLTs, as well as a definite localization of VWF in the PLT membrane. Interestingly, S-GBM-PLTs showed the ability to form VWF fibers, a widely recognized marker of activation (Figure 2, column B). The quantification of the MFI revealed that NS-GBM-PLTs expressed more intra-PLT VWF (Figure 3E), while a significantly more intense signal was detected in S-GBM-PLTs after stimulation compared to HDs (Figure 3F). A similar tendency was detected for S1P-related factors, such as S1P, Sphk1, S1PR1, and SPNS, both under basal conditions and after stimulation. Indeed, especially for S1P, a strong signal was visible inside GBM-PLTs in the NS condition together with S1PR1 (Figure 2, column C, Figure 3G–I). When stimulated, GBM-PLTs released S1P in the extracellular milieu, thereby enhancing S1P labeling outside PLTs (Figure 2, column C, Figure 3H). Similar behavior was shown by S1PR1, which was localized in the cell membrane in NS-GBM-PLTs and released after activation, probably via microvescicle-mediated secretion (Figure 2, column C, Figure 3I). For SphK1 and SPNS, enhanced fluorescence was observed after stimulation of GBM-PLTs (Figure 2, column D and column E, respectively), both in terms of intra-PLTs and the releasate. The quantification of MFI in Sphk1-positive PLTs positive revealed similar expression levels in HDs compared to GBM patients, both under the resting and stimulated conditions (Figure 3J). Interestingly, GBM-PLTs showed greater expression for SPNS compared to HD-PLTs (Figure 2, column E); this increment was statistically significant for the stimulated PLTs (Figure 3K). The immunopositivity for P-selectin showed a significant increment in GBM-PLTs after stimulation compared to HD-PLTs (Figure 3L).

### 3.3. Protein Content in GBM-PLTs 

**Figure 4:**
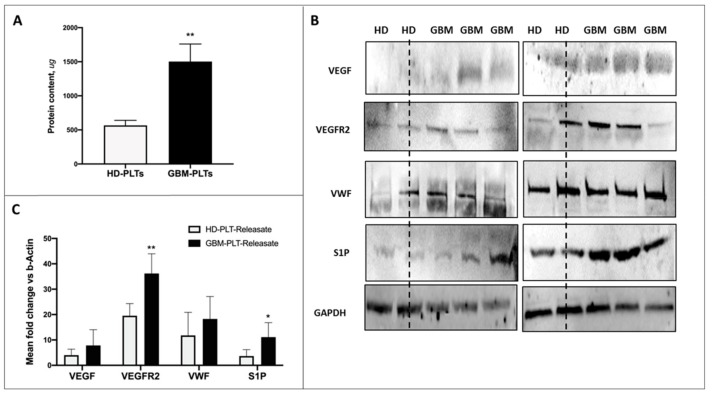
Pro-angiogenic and pro-tumoral protein contents in GBM- and HD-PLTs. (**A**) Quantification of HD- and GBM-PLT protein contents. Data are shown as mean ± SD of all samples analyzed, normalized to the PLT count. (**B**) Densitometric quantification of the protein expressions of VEGF, VEGFR2, VWF, and S1P in all of the analyzed HD- and GBM-PLTs. Data were normalized to GAPDH expression, used as an endogenous control, and reported as the mean ± SD. (**C**) Representative images of Western blot analysis for the assessment of protein concentrations of VEGF, VEGR2, VWF, and S1P in PLT-releasate from 2 HD-PLTs and 4 GBM-PLTs. Notably, the graph in Figure 4C reports densitometric data versus GAPDH. * *p* < 0.05, ** *p* < 0.01, *** *p* < 0.001 versus healthy donors (HD).

Quantification of protein content revealed that, when normalized to the PLT count, a greater amount of protein was present in GBM-PLTs compared to HD-PLTs (Figure 4A), indicating the ability of GBM-PLTs to act as active carriers of potentially tumor-derived oncopromoter mediators. The investigation of the expression of pro-angiogenic factor by Western blotting exposed GBM-PLTs to contain higher concentrations of VEGF, VEGFR2, VWF, and S1P (Figure 4B). The quantification of densitometry revealed that, when normalized to GAPDH expression, VEGF, VEGFR2, VWF, and S1P were 2.5-, 2.1-, 2.4-, and 5.8-fold higher, respectively, in GBM-PLTs than in HD-PLTs (Figure 4C).

### 3.4. GBM-PLT-Releasate Enhances Angiogenesis in GECs

**Figure 5:**
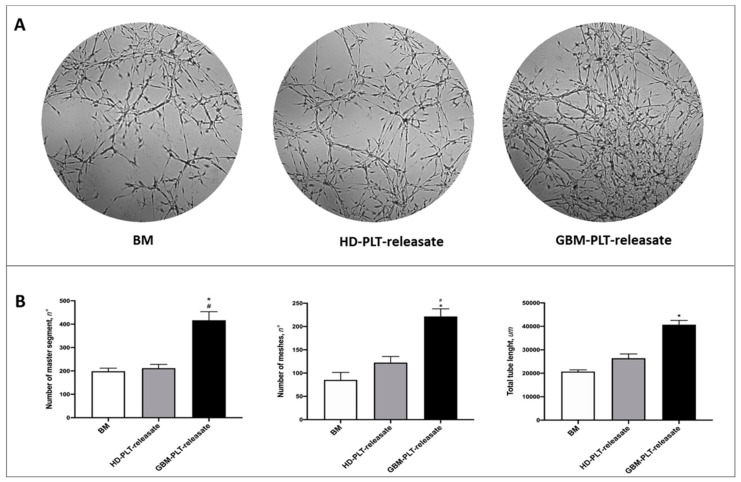
GBM-PLT-releasate strongly induced GBM endothelial cell (GEC) cord formation. (**A**) Representative images of GECs cultured for 24 h in Matrigel with PLT-releasate from HD- and GBM-PLTs for 24 h. (**B**) Quantification of number of master segment meshes and total tube length measured using the Angiogenesis Analyzer plugin in ImageJ. Data are the mean ± SD of at least 3 experiments in triplicate. * *p* < 0.05 versus basal medium (BM); ^#^ *p* < 0.05 versus HD-PLT-releasate.

The tube-like structure formation displayed the pro-angiogenic effect of the releasate of GBM-PLTs on GECs. Indeed, in the presence of GBM-PLT-releasate, GECs increased the number of “vessel-like” cords, segments, and meshes, thus creating a more complex vascular network. On the contrary, only a slight effect was observed when GECs were cultured in the presence of HD-PLT-releasate (Figure 5).

## 4. Discussion

Over the last few decades, an increasing number of clinical and experimental studies investigated the association between PLTs and tumorigenesis, as well as drug resistance. Indeed, PLTs may serve as circulating carriers of pro-tumor factors, thus acting systemically and locally in a beneficial manner regarding tumor progression and stimulating tumors to be resistant to therapies [23]. For this reason, unraveling the functional contribution of PLTs in tumor angiogenesis and growth may potentially provide better strategies for cancer treatment [24]. In this work, we hypothesized that the highly intense hypervascularization of GBM contributes to an overexposure of VWF on the GEC surface, thereby determining the massive recruitment and activation of circulating PLTs. At the site of injury, tumor cell-induced platelet activation (TCIPA) is characterized by PLT aggregation, adhesion, and a strong increase of PLT-derived pro-angiogenic factors [25]. PLTs are activated by their essential pathways, such as thromboxane (TX)-A2, glycoprotein (GP)-Ib-IX, ADP, and GPIIb/IIIa [26], leading to a dramatic increase in PLT-activation markers, such as P-selectin (CD62P) and angiogenesis mediators. Literature evidence reported that PLTs from breast cancer patients contained significantly higher levels of VEGF, angiopoietin-1, and P-selectin compared to normal controls [27]; similarly, the concentrations of VEGF and platelet-derived growth factor (PDGF) were increased in colorectal cancer patients compared to controls [28], as well as in GBM patients [20]. In this study, we asked whether soluble factors released from activated TEPs could influence GBM aggressiveness, thereby ensuring growth factor bioavailability and promoting angiogenesis and thus shifting the tumor microenvironment in favor of cancer progression. Our results revealed that GBM-PLTs present an activated state, proved by the expression of P-selectin, compared to HD-PLTs. This activation status increases after in vitro stimulation, which mimics the typical TCIPA occurring in GBM and, in particular, in the GBM vascular network. TCIPA induces the fine mechanism of “tumor education”, by which PLTs fill up with pro-angiogenic factors, which are in turn secreted after TCIPA in a self-sustaining vicious cycle. Immunolabeling showed the different activation states of HD- and GBM-PLTs, revealing a higher expression of activation markers, pro-angiogenic, and oncopromoter factors, such as P-selectin, VEGFR1, VEGFR2, VWF, S1P, its receptor S1PR1, the kinase SPhK1, and the transporter, SPNS2. Likewise, the investigation of PLT-releasate after induced activation revealed that GBM-PLTs carried higher amount of the same oncopromoter mediators, such as S1P, VWF, VEGF, and VEGFR2. PLTs are able to produce and store large amounts of S1P, and the biosynthetic process guided by SphKs represents the major source of S1P production [29]. However, although they possess high SphK activity, PLTs lack the *de novo* sphingolipid biosynthesis necessary to provide substrates. Interestingly, PLTs are able to incorporate extracellular 3H-labeled Sph much faster than human megakaryoblastic cells, and rapidly convert it to S1P [30]. Therefore, the high concentration of S1P into PLTs is determined by two sources: generation by extracellular Sph incorporation and phosphorylation by intraplatelet SphKs, and storage during PLT biogenesis from megakaryocytes (MKs), as SphK messenger RNA (mRNA) was reported to be the major Sphk transcript expressed in MKs [14]. Furthermore, it was demonstrated that the serum level of S1P increased about 2-fold after clot formation, suggesting that PLT aggregation led to S1P release [31]. Indeed, experimental evidence reported that PLT activation could strongly augment local S1P concentration, potentially engaging receptors or shifting the balance regarding their activation. The secretion of S1P in the extracellular milieu may be mediated both by the S1P transporter SPNS and PLT degranulation. Therefore, the increase in S1P concentration in stimulated PLTs as well as in PLT-releasate perfectly matches with the higher activation status and with the overexpression of SPNS. Thanks to the expression of S1P receptors on vascular smooth muscle cells, the increase in the S1P concentration plays a key role in endothelial repair by switching on the angiogenic mechanism. Similarly, in GBM, the local increase in S1P by PLT release after TCIPA, exerts a pro-angiogenic stimuli for neovascularization, thereby allowing the formation of pathological blood vessels which provide nutrients and oxygen for tumor growth. From this evidence, it is reasonable to explain the higher positivity of Sphk1 and S1PR1 in activated GBM-PLTs compared HDs, therefore a key contribution of PLT-derived S1P in GBM progression can be hypothesized. Likewise, VEGF and its signaling pathway are the most important positive regulators of angiogenesis, acting as an effective mitogen and survival factor for GECs and impacting blood vessel integrity and permeability. The high expression of VEGF and its receptors was widely demonstrated in GBM and many other forms of cancer, leading to a monoclonal antibody to be developed against VEGF, bevacizumab (Avastin^®^), which is administered for colorectal cancer, metastatic breast cancer, and non-small cell lung cancer, as well as for relapsed forms of GBM [32]. It was demonstrated that the local high concentration of VEGF was supported by PLT contribution, as PLTs store VEGF in their α-granuli, which are released after TCIPA. We previously reported that the intraplatelet concentrations of VEGF were significantly elevated in primary GBM, and ADP- and thrombin-stimulation led to high levels of VEGF in the PLT-releasate, which we found to be directly correlated with patient survival in our study group [20]. It is now clear how essential this much higher expression of VEGF and VEGFR1/2 in GBM-PLT is in terms of VEGF carrying, both at the local and systemic levels. In GBM, VEGF-mediated neovascularization leads to the formation of fenestrated blood vessels, subsequently producing a loss of integrity and increased permeability. The strong activation of GECs causes exposition on their surfaces for VWF, which mediates PLT recruitment, aggregation, and activation. We previously reported higher plasma levels of VWF antigen (VWF:Ag) in GBM patients compared to patients with benign lesions, showing that higher VWF plasma levels were correlated with shorter survival, rendering VWF:Ag levels a putative circulating prognostic factor [11]. In this work, we demonstrated that VWF is a component of PLT cargo and is released in the GBM tumor mass after TCIPA, thereby supporting a self-sustaining vicious cycle. Finally, we tested the effect of this enriched PLT-releasate on GEC angiogenic potential via the tube-formation assay. The results revealed a strong effect of PLT content on GECs, which managed to form a complex vascular network with an increased number of cords and meshes.

**Figure 6:**
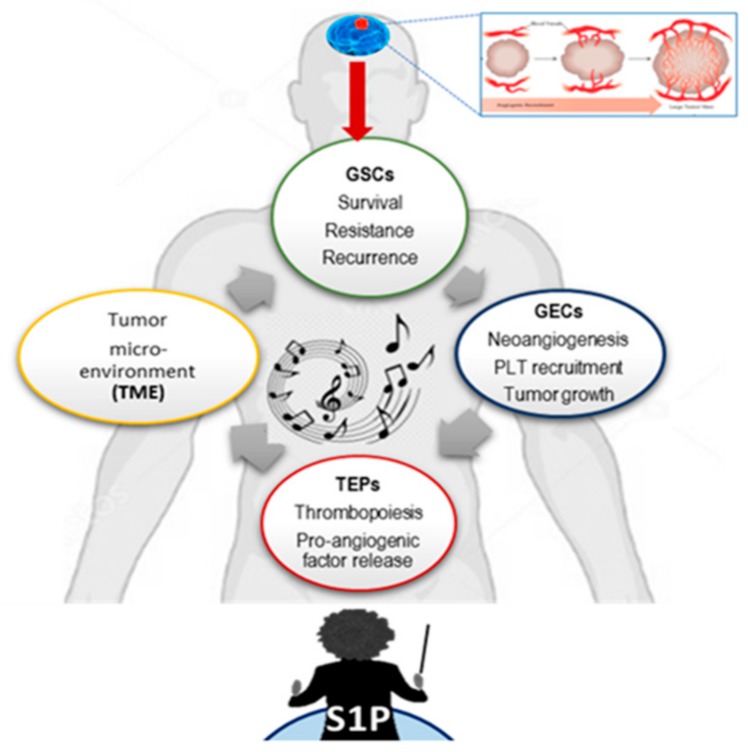
The concert between GBM stem cells (GSCs), GECs, PLTs, and tumor microenvironment (TME), orchestrated by S1P. A schematic representation of the concept of a self-sustaining vicious cycle, in which, in “CONCERT”, S1P plays the master regulator role, acting similar to an “orchestra’s” conductor, telling the ”musicians”, i.e., GSCs, GECs, PLTs, and VWF, who and when should play in the complex, multi-factorial “GBM symphony”. This theory fills the gap between the local and systemic key events, changing the vision of GBM from a localized tumor confined to the brain to a systemic pathology, in which many different organs and cell subpopulations give their contribution via S1P-orchestated mechanisms.

Taken together, our findings support the concept of a self-sustaining vicious cycle, in which TEPs are considered to be local and systemic responders to the presence of cancer, sequestering, carrying, and releasing tumor-derived biomolecules as RNA, proteins, and especially bioactive lipids, such as S1P, thereby supporting tumor progression. This work investigated the role of sphingolipid signaling/metabolism in gliomagenesis, filling the gap between local and systemic events and changing the vision of GBM from a localized tumor which is confined to the brain to a systemic pathology, in which many different organs and cell subpopulations contribute via S1P-orchestated mechanisms (Figure 6).

## 5. Conclusions

GBM is the deadliest primary brain cancer in adults. Despite the development of different therapeutic approaches, limited progress has been made to improve the poor patient overall survival, suggesting that novel paradigms should be considered. Drug resistance also remains a problematic challenge for both scientists and clinicians. Due to their strong association with tumors via tumor education and TCIPA mechanisms, activated PLTs with their oncopromoter cargo potentiate chemoresistance of cancer cells through the regulation of tumor growth and angiogenesis. Therefore, targeting the two-way signal exchange between tumors and PLTs and, more specifically, targeting PLT-originated growth factors may represent a promising approach to enhance the sensitivity of tumors to therapy. Finally, PLTs may be used as easily accessible, non-invasive biomarkers to predict the response to therapy and monitor tumor progression and eventual relapses via a personalized medicine strategy.

## Figures and Tables

**Table 1:** Demographic, clinical, and molecular parameters of the patient cohort.

Patient ID	Sex	Age	Diagnosis	KPS (%)	IDH	MGMT (%)	Ki-67 (%)	Tumor Region
**HD01**	F	28	N/A	-	-	-	-	-
**HD02**	F	39	N/A	-	-	-	-	-
**HD03**	M	37	N/A	-	-	-	-	-
**HD04**	F	53	N/A	-	-	-	-	-
**Poli 201**	F	60	GBM	80	*w*/*t*	2	20	PO sx
**Poli 202**	F	68	GBM	80	*w*/*t*	34	70	T dx
**Poli 203**	M	69	GBM	80	*w*/*t*	25	40	TP dx
**Poli 204**	M	67	GBM	80	*w*/*t*	8	20	TP dx
**Poli 205**	M	49	GBM	90	*w*/*t*	32	12	PO dx
**Poli 206**	M	73	GBM	70	*w*/*t*	16	25	PO dx

M: male, F: female; KPS: Karnofsky performance status; IDH: isocitrate dehydrogenase; MGMT: O (6)-methylguanine-DNA methyltransferase; PO: parietal–occipital region, T: temporal region, TP: temporal–parietal region.
